# How can we improve the timeliness and quality of diagnostic assessment for children with possible autism? Qualitative findings and recommendations from a Realist Evaluation of Autism Service delivery in the United Kingdom

**DOI:** 10.1177/13623613261430914

**Published:** 2026-05-10

**Authors:** Ian Male, William Farr, Sophie McGrevey, Vanessa Abrahamson, Sarah Wigham, Venkat Reddy, Amanda Allard, Victoria Grahame, Jessica Maxwell, Grainne Saunders, Anna Walker, Nic King, Seema Islam, Zamir Akhtar, Jeremy Parr, Patricia Wilson

**Affiliations:** 1 Sussex Community NHS Foundation Trust, UK; 2 Brighton and Sussex Medical School, UK; 3 University of Kent, UK; 4 Population Health Sciences Institute, Newcastle University, UK; 5 Cambridgeshire and Peterborough NHS Foundation Trust, UK; 6 Council for Disabled Children, UK; 7 Cumbria, Northumberland, Tyne and Wear NHS Foundation Trust, UK; 8 West Sussex Parent Carer Forum, UK; 9 Independent Advocate, UK; 10 Newcastle upon Tyne Hospitals NHS Foundation Trust, UK

**Keywords:** autism, children, diagnosis

## Abstract

Families face long waits for their child to undergo a diagnostic assessment for possible autism. This study explored the experiences of parents and children/young people (CYP) accessing and professionals providing autism diagnostic assessments in the United Kingdom. This realist evaluation was conducted in three phases: (1) interviews and focus groups with parents, CYP and professionals from six U.K. National Health Service (NHS) childhood autism assessment and diagnosis services; (2) resulting qualitative data were analysed, and programme theories (PTs) explaining how, why and in what contexts childhood autism assessment and diagnosis pathways work well were tested and refined; and (3) recommendations for change were developed by the research team and presented to delegates at six dissemination events including 250 clinicians, managers, parents/carers, commissioners and academics involved in autism assessment and diagnosis. Delegates were invited to select and rank the most important recommendations. There were 121 participants in Phase 1 (18 CYP, 34 parents, 69 professionals). In Phase 2, both families and clinicians confirmed challenges in the assessment and diagnostic process, including increased demand for assessments and insufficient numbers of specialist multidisciplinary team (MDT) practitioners to provide adequately skilled workforces. The need to support families across the whole assessment and diagnostic process was seen as key to providing a quality service but was often not possible. Steps taken to improve the process were identified, and seven PTs covered: improving recognition of children needing referral, referral processes, service organisation, professional skill mix, assessment, feedback/report writing and training/service evaluation. In Phase 3, during consultation, 12 recommendations were selected as most important for change. Several strategies for improving services were identified that may reduce waiting times and increase acceptability and quality of childhood autism assessment and diagnosis services.

**Lay Abstract:**

Waiting lists for childhood autism assessments are lengthy, meaning families wait a long time for their child to undergo a diagnostic assessment for possible autism. In this study, we explored the experiences of children, young people and parents who have gone through the assessment and diagnosis process in the United Kingdom. We also explored the views of professionals who deliver childhood autism diagnostic assessments. We conducted interviews and focus groups with children, young people, parents and professionals. We recruited participants from six U.K. National Health Service (NHS) assessment and diagnosis services. We wanted to find out how childhood autism assessment and diagnosis processes could be improved. We asked the research participants about their experiences of the autism assessment and diagnosis process to find out what worked well, who it worked well for and under what circumstances. Recommendations for change were developed by the research team and presented to delegates at six dissemination events. Delegates (including 250 clinicians, managers, parents/carers, commissioners and academics involved in autism assessment and diagnosis) were invited to select and rank the most important recommendations. In total, 121 people took part including 18 children and young people, 34 parents and 69 professionals. Participants described their experiences of the assessment and diagnosis process and challenges in the system including increased demand for assessments and there not being enough specialist skilled practitioners available to conduct assessments. Participants also described ways the childhood assessment and diagnosis process could be improved, including better information gathering during referral. The need to support families throughout the whole assessment process (and not just on receiving a diagnosis) was seen as very important. Seven key areas for improvement were identified: accurately recognising when to refer children, the referral process, service organisation, skill mix of autism assessment teams, the diagnostic assessment, feedback and report writing, and training for staff. Twelve recommendations for change were identified by delegates at the dissemination events. The findings can be used to help make recommendations for service development, reduce waiting times and improve the quality of childhood autism assessment services for children, young people and families.

## Introduction

Waiting times for assessment of possible autism in children have increased significantly in recent years in the United Kingdom and continue to rise (Morris, 2024), leading to calls for service delivery solutions to reduce waiting times and improve assessment quality (NHSE, 2019; UKGov., 2024). Research in the United Kingdom shows referral rates doubled between 2015 and 2019 (Parr et al., 2024), with a fivefold increase since 2019 (Morris, 2024), and a 787% increased incidence of autism diagnosis between 1998 and 2018 (Russell et al., 2022). In 2023, the median wait time for an initial assessment was 9 months (Morris, 2024), although reaching diagnostic conclusions and formulation may take over a year (Male et al., 2020). The delays in accessing an autism assessment is of relevance in many other countries too including the United States (Gordon-Lipkin et al., 2016; Kanne & Bishop, 2021; Williams-Arya et al., 2019), Canada (Penner et al., 2018; Smith-Young et al., 2020), Europe (Bejarano-Martín et al., 2020) and increasingly Asia (Rah et al., 2020; Sasayama et al., 2021). Delays to diagnostic assessment can reduce timely access to interventions and support, potentially negatively impacting outcomes for children and families. Without accurate diagnosis, many children struggle at school or home and may develop mental health problems or end up in crisis or even inpatient care (McGuire et al., 2015).

In the United Kingdom, evidence-based guidance recommendations and quality standards for assessment and diagnosis of autism are published by the National Institute for Health and Care Excellence (NICE, 2011, 2014). Diagnostic assessment generally requires input from a multidisciplinary team (MDT) (NICE, 2011), with significant human and financial resourcing; our research showed a typical assessment takes 11–14 hr of professional time, costing £8–900 (Galliver et al., 2017; Male, Farr, Bremner, et al., 2023). In the United Kingdom, such assessments can be carried out by three main NHS service types or pathways: Child Development or Paediatric Teams (CDTs), Child and Adolescent Mental Health Services (CAMHS), or in some cases integrated pathways which combine CAMHS and CDT services, the latter broadening the disciplines available to the MDT (Male, Farr, Allard, et al., 2023; Parr et al., 2024). Services can be autism-specific or neurodevelopmental, where parallel assessment pathways are provided for autism and/or other neurodevelopmental conditions, for example, attention deficit hyperactivity disorder (ADHD). Several approaches have been identified to improve access to timely diagnosis, for example, improving referrers’ knowledge (Gordon-Lipkin et al., 2016), developing MDT skill mixes, offering support early in the process rather than waiting for diagnosis and operating an holistic neurodevelopmental approach rather than focussing on a single diagnosis such as autism (Abrahamson et al., 2021; Parr et al., 2024).

This article presents qualitative findings and recommendations from a wider research programme (Realist Evaluation of Autism ServiCe Delivery [RE-ASCeD]) exploring how to improve timeliness and quality of childhood autism assessment and diagnosis pathways. Realist evaluation enables understanding complex interventions including service delivery (where there is rarely a one-size-fits-all solution) and what factors (contexts) trigger mechanisms that facilitate, or impede, the success of an intervention (outcome) (Pawson & Tilley, 1997). These relationships can be represented as Context-Mechanism-Outcomes (CMO) configurations or programme theories (PTs), explaining how, why and in what contexts a programme/intervention is successful or not (Pawson & Tilley, 1997). These are often presented as ‘If . . . Then’ statements, for example, it may be that service configuration, for example, co-location of CAMHS and CDTs (Male et al., 2020) or the needs of an individual child and family, such as complexity of diagnostic presentation (Penner et al., 2023; Whitehouse et al., 2018), can impact on outcomes.

Prior to this study, the RE-ASCeD research programme included a Rapid Realist Review (RRR) (Abrahamson et al., 2020, 2021) and a survey of U.K. NHS childhood autism assessment and diagnosis services (Parr et al., 2024). The review and survey findings identified challenges to childhood autism diagnostic pathways including increasing demand for assessments, difficulties with staff recruitment and retention, disjointed service provision and increased case complexity (Abrahamson et al., 2021; Parr et al., 2024). The RRR and survey contributed to developing initial programme theories (IPTs) describing factors that may contribute to timely and high-quality childhood autism diagnostic pathways. These PTs covered initial recognition of possible autism, referral and triaging, diagnostic model, feedback to parents, working in partnership with families, interagency working, and training and service development (Abrahamson et al., 2021).

The aim of the current study was to iteratively test and refine the initial PTs based on the views of parents, children/young people (CYP) and professionals with experience of autism diagnostic services and investigate which approaches facilitate autism diagnostic services offering timely, high-quality and acceptable assessments for children and families. In line with realist thinking, a further aim was to identify contexts and mechanisms impacting success or failure of these approaches if implemented in other provider contexts and from this to make recommendations over how to improve service delivery in the future.

## Method

The study was conducted in three phases. In Phase 1 CYP, parents and professionals from six U.K. NHS childhood autism diagnostic services took part in interviews or focus groups. Phase 2 was conducted in parallel to Stage 1 with data collected in Phase 1 used to test and refine the seven PTs, explaining how, why and in what contexts childhood autism assessment pathways work well. In Phase 3, the PTs were developed into recommendations in consultation with childhood autism assessment pathway service users/parents, practitioners and service providers. Inclusion criteria were children aged ⩽16 years entered into, or already in, an autism diagnostic pathway and under the care of the participating NHS diagnostic teams and parental consent obtained.

### Setting and participants

Sites were purposively selected from those participating in our U.K. survey (Parr et al., 2024). Criteria for selection were (1) reporting providing services largely compliant with NICE guidelines (NICE, 2011, 2014) and (2) representative of different U.K. service types: CDTs, CAMHS and integrated services (please see description of service types in section ‘Introduction’). Participating sites included two CDTs (CDT 1 and 2), two CAMHS (CAMHS 1 and 2) and two integrated services with CDT and CAMHS working together (Integrated 1 and 2). Two services operated a neurodevelopmental pathway (NDP) (CAMHS 2, Integrated 2), while Integrated Service 1 provided assessment of autism and ADHD but under separate pathways. The remaining teams operated autism-specific pathways.

Individual participants were selected by site investigators using purposive sampling, facilitating representativeness in terms of age, gender and ethnicity of CYP and type of MDT professional. Potential CYP participants were provided with study information by the local MDT; those who expressed interest in participating were then contacted by the research team for informed consent. Professionals were approached directly by the research team and provided with study information. In total, 126 participants were recruited for qualitative interviews, of whom 121 (18 CYP, 34 parents, 69 professionals) participated (Tables 1 and 2). Five participants withdrew due to time constraints.

**Table 1.** Characteristics of CYP and parents.

Site	Parents interviewed (*n*=)	Mother/father	Gender (male/female)	Ethnicity (W: White; B: Black; M: mixed)
Integrated 1	8	8/0	0/8	3W, 2B, 3M
Integrated 2	6	5/1	1/5	6W
CDT 1	4	4/0	0/4	2W, 2M
CDT2	6	6/0	0/6	6W
CAMHS 1	4	4/0	0/4	3W, 1B
CAMHS 2	6	5/1	1/5	6W
Site	CYP interviewed	Mean age (years) at time of interview (range)	Gender (boy/girl)	Ethnicity
Integrated 1	6	10.4 (6-14)	5/1	4W, 1B, 1M
Integrated 2	2	12 (12)	1/1	2W
CDT 1	0	-		
CDT 2	4	12.7 (10-14)	2/2	4W
CAMHS 1	6	16 (13-18)	4/2	3W, 1B, 2MV
CAMHS 2	0	-		

MV: missing value; CYP: children and young people; CDT: Child Development Team; CAMHS: Child and Adolescent Mental Health Services.

**Table 2.** Professionals recruited.

Site	1 Integrated	2 Integrated	1 CDT	2 CDT	1 CAMHS	2 CAMHS
Professional Role						
Paediatrician	1	2	3	4	3	1
Educational Psychologist	1	0	0	0	0	0
Clinical Psychologist	3	4	2	0	1	0
Assistant Psychologist	1	0	0	0	0	0
SENCo	2	0	1	0	0	0
SALT	1	0	1	2	5	0
Health Referrer	1	0	0	2	0	0
Manager	1	2	3	0	2	2
Commissioner	0	2	0	0	0	1
Support Worker	0	2	0	0	0	0
CAMHS Practitioner	0	1	0	0	0	0
Occupational Therapist	0	0	1	0	1	2
Specialist Nurse/Health Visitor	0	0	1	0	0	1
Child Psychiatrist	0	0	0	0	1	0
Portage Worker	0	0	0	2	0	0

SENCo: Special Educational Needs Coordinator; SALT: speech and language therapist; CAMHS: Child and Adolescent Mental Health Services; CDT: Child Development Team.

**Figure 1.**
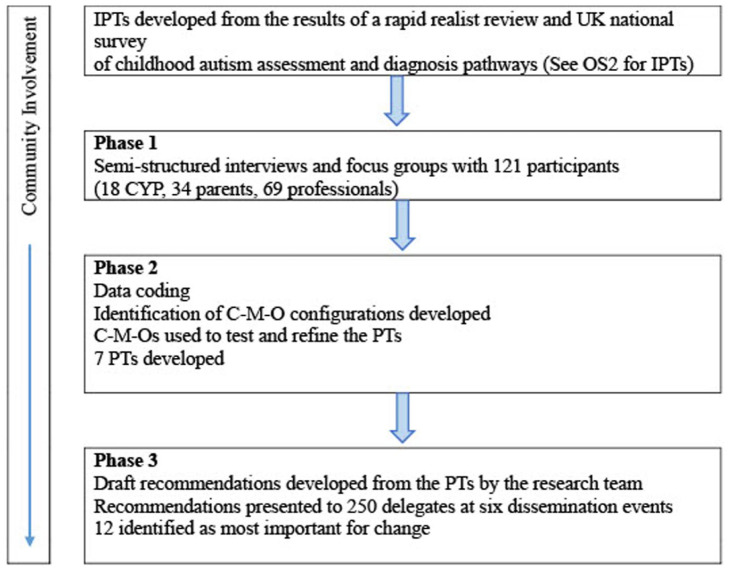
Flow chart of the relationships between research stages and the development of the programme theories and recommendations.

Draft recommendations developed by the research team, questions and conclusions (Supplementary OS 3 Figures 1 to 3) were presented to delegates at six dissemination events including two Continuing Professional Development (CPD) workshops at the British Association of Community Child Health annual conference; two CPD workshops at the Council for Disabled Children (CfDC) conference; one Parent and Expert Advisory Committee event; and one national consensus event hosted by CfDC.

The study was given a favourable opinion by the London Central Research Ethics Committee and the Wales Health Research Authority (Reference: 21/LO/0084) on February 3, 2021.

### Data collection and analysis

#### Phase 1

Semi-structured interview and focus group schedules were developed and informed by our RRR and U.K. national survey findings (Abrahamson et al., 2021; Parr et al., 2024) and piloted with research and clinical colleagues, parent co-investigators, and a CYP Patient and Public Involvement and Engagement (PPIE) group. Topic schedules were developed for each participant group and are shown in Supplementary Table OS1A to D. These were designed to gather data to test IPTs. A table showing the IPTs referred to as a guide for interviewers during the interviews and focus groups is shown in Supplementary Table OS2. All interviews and focus groups were conducted via Microsoft Teams, audio-recorded and transcribed. Interviews were conducted by research assistants/associates, each responsible for two NHS sites. CYP interviews lasted around 30 min, based on prior research and advice from the CYP PPIE group, who were also consulted regarding interview accessibility where required. Professional interviews lasted 30–60 min, and interviews and focus groups for parents lasted 30–90 min. Researchers made notes during interviews to aid interpretation of transcribed recordings.

#### Phase 2

There were 121 transcripts from the interviews and focus groups totalling approximately 2,000 pages, approximating to 100 hr of interviews. Researchers responsible for data collection coded transcripts from their two sites, and all data were second-coded by one team member (P.W.). Coding was recorded in Microsoft Word.

Forty-eight transcripts (43%) (13 focus groups, 7 CYP interviews and 28 professional interviews) were read and discussed by research team members (P.W., V.A., S.M., I.M., W.F., S.W.) at monthly online transcript discussion meetings to agree emerging themes, identify CMO configurations and make comparisons across different sites and service types. All members coded agreed transcripts and reflexively discussed key quotes, emerging themes and how they related to the pre-existing PTs. These were discussed with the wider team to agree main findings and resulting PTs. Themes and CMOs were used to test and refine the IPTs in an iterative process. After several transcript discussion meetings, saturation point was reached with no further information or insights emerging (Nelson, 2017) and the wording of the final 7 PTs agreed.

#### Phase 3

The research team met to discuss Phase 2 findings and reach agreement over summarising the seven PTs into recommendations for improving timeliness and quality of childhood autism diagnostic service delivery. The recommendations were presented to attendees at six dissemination and consultation events during PowerPoint presentations. During consultation, we did not gather information about participants’ personal characteristics, and they were not required to consent in this consultation-based phase. Six key questions and summary conclusions developed from the PTs were presented on slides to attendees at the dissemination events to set the scene for selecting the recommendations viewed as most important for change (Supplementary Figures 1 to 3 OS3). Attendees were invited to discuss the recommendations and select the most important in the context of their experiences. This included challenges and solutions and providing written feedback including ranking of the recommendations. Some delegates participated, and others chose not to. The feedback from attendees and their ranking of the recommendations led to the identification of the 12 most important for change and service improvement.

The relationships between the research stages and the development of the PTs and recommendations are shown in Figure 1 and Table 3.

**Table 3.** Process of developing the PTs.

Procedure	Final programme theories
121 transcripts from the interviews and focus groups Transcripts coded Portion of transcripts double-coded Transcripts and coding discussed in team meetings CMO configurations identified CMOs and developing PTs discussed in team meetings 7 final PTs defined	1: Improve Recognition of Children Needing Referral
	2: Improve Quality of Referrals
	3: Service Organisation
	4: Skill Mix and Effective Team Working
	5: Approaches to Improve Assessment
	6: Diagnostic Feedback and Reporting
	7: Training, service development and evaluation (Maintaining and Growing the Workforce)

### Participatory methods and positionality

Parents and charities representing those with lived experience (CfDC, Autistica) contributed throughout the study during consultation, including advising the researchers on study design, data analysis, interpretation of results and dissemination. Community involvement included a CfDC Parent and Patient Partnership group, consisting of four parents of autistic children, who met monthly with one of the Chief Investigators, to review and co-produce study documents, interview schedules and to review a selection of transcripts during data analysis. Emerging findings and PTs were also reviewed with the community involvement representatives, and issues were fed back to the research team. The experience of this group is reported elsewhere (Farr et al., 2022). One parent representative was a core research team member. Findings were also discussed with an Expert Advisory Group bringing a mix of clinical and academic expertise on autism assessment; members included contributors to the development of the NICE guidelines.

The coding team comprised six researchers (four females, two males: experienced in qualitative research, autism research and trained in Realist Evaluation) led by a realist evaluation expert (P.W.) and a paediatrician expert in autism assessment and diagnosis (I.M.). Regular consultation with the Parent and Patient Partnership group and the Expert Advisory group ensured research team decisions were informed by feedback and advice from people with lived experience and clinical academic experts throughout the study and facilitated the researchers examining their own views and perspectives reflexively.

### Data availability statement

Some data from this study are available on reasonable request from the corresponding author. Data about services provided by named NHS Trusts will not be shared.

## Results

### Phase 2

During an iterative process of testing and refining, seven PTs were constructed: recognition, referral, service organisation, skill mix, assessment, feedback/reporting and training/evaluation. Compared with the original PTs, this final iteration split ‘diagnostic model’ into service organisation, skill mix and assessment. Working in partnership with families, providing support, and effective interagency working, were considered important at all stages of the diagnostic process so overarching and incorporated into each PT, rather than stand-alone PTs. A summary of the PTs is shown in Supplementary OS4; quotes describing each PT are shown below (with participant and service type in brackets) and in Supplementary OS5.

#### PT1. Improve recognition of children needing referral

Many parents interviewed described years of stress dealing with their child’s difficulties and not being listened to when raising concerns. Parents talked about having to jump through hoops before they were eventually seen and often thought it was more luck than system-wide awareness that professionals responded to their concerns:

. . .we’d obviously had a lot of challenges with (x) for a lot of his life and I’d gone back and forth to primary school a lot of times [Integrated 2 Parent]

There were exceptions to parents not feeling listened to, particularly for pre-school children with clear presentations of autism when parents’ concerns were acted upon or the possibility of autism raised by professionals:

I think the nursery listened to our concerns. I’d spoken to the health visitor about the fact he wasn’t talking., I . . . relayed my concerns to them and they listened. And then, everything kind of just snowballed from there and got him diagnosed . . . they really listened and then obviously got the help that he needed . . . People listening to you and understanding what’s going on definitely helped. [CDT 2 Parent]

The consequence of concerns being repeatedly disregarded included delayed referrals for diagnostic assessment, parents feeling blamed for making too many claims about their child’s needs and young people feeling they were the problem:

It was in primary school. It was mostly it went undetected, so I always thought the problem was my attitude . . . I always felt like I was the problem, and I felt like I needed to change something about myself. [Integrated 1 CYP]

Where there were significant delays in assessment, this impacted negatively on parent’s perceptions of the whole process, even where acknowledging the diagnostic team was helpful once involved. Several interviewees reported that health visitors (HVs) and general practitioners (GPs) varied in knowledge and skills in recognising potential autism. Some sites responded to this by providing training to community colleagues. In Integrated Site 2 and CDT 2, HVs undertook home assessments as part of the overall process, shortening the time needed for assessments. Parents from these sites reported their concerns were listened to and acted upon quickly by frontline staff. CDT 1 employed a specialist HV whose role included upskilling generic HVs to recognise possible autism and facilitate referrals.

Parents reported differing experiences of schools’ awareness regarding autism. Some schools were described as responding to the child’s needs rather than only taking note of issues if a diagnosis was given; others recognised the child’s needs but had inadequate resources to meet them. The perception that support could only be offered for children with a diagnosis sometimes acted as a driver for referral, with some schools suggesting diagnosis was necessary to apply for an Education, Health and Care Plan (EHCP) (a formal document specifying a child’s additional support requirements that must be provided by local services).

#### PT2. Improving referral quality

All teams experienced significant increases in referrals: ‘when [the service] was formed it was . . . to see about 60 children a year, we now think it’s probably about tripled’ (Integrated 1 Educational Psychologist). Waiting lists were challenging for providers and parents. Solutions included prioritising some children with triage based on information gathered at referral, for example, those at risk of permanent school exclusion or with flexible/tiered assessments responsive to the complexity of child and family characteristics.

Parents often thought referral quality was poor, leading to delays in being accepted. While GPs often initiated referrals, they were not familiar enough with the child to provide sufficient information:

. . . GPs don’t know them . . . we appreciate they don’t have time, because all they have is ten minutes, so the referral isn’t as good. [CDT 1 Consultant Paediatrician]

When referrals took a long time, parents frequently reported information was outdated. Where there were separate pathways and referral routes for individual conditions (e.g., autism, ADHD), referrals were commonly passed back-and-forth (‘bounced’) between pathways, lengthening the wait for acceptance/refusal and for assessment to start. Some sites utilised a Single Point of Access (SPOA) for referrals, a gateway to the assessment process offering one clear pathway and ‘no wrong door’. When CYP were referred to the ‘wrong’ team, the SPOA directed the referral to the correct pathway, ‘rather than rejecting the referral and asking the GP to make another one to the right service’ (CAMHS 1 Team Lead).

Receiving a detailed referral describing the child’s strengths and needs allowed teams to operate a more flexible assessment approach. At Integrated Site 2, children underwent strengths and needs assessments, parents received support from the Early Help Service, and referrals were only accepted once this was completed. The team reported this reduced the number of families subsequently requesting diagnostic assessment by around 30%. However, some parents thought it delayed access to diagnostic assessment. The information obtained, coupled with this integrated service running a neurodevelopmental assessment model, enabled a child with suspected autism and ADHD to undergo a joint assessment with professionals able to consider both conditions within a single pathway.

Finally, having professionals with the skills and tools to accurately describe relevant child characteristics in a referral was considered important. Teams addressed this through improving information collection methods, such as open-text questionnaires to gather initial information describing the child from school and parents, while CDT Site 1 created a Special Educational Needs and Disabilities (SEND) HV role to add relevant details to incomplete referrals. Another site described,

. . . if there was (an) incomplete referral mainly from GP services, I would then take that referral back and offer the parent a . . . social communication questionnaire and usually do that so that it’s quicker. [CDT 1 SEND HV]

#### PT3. Service organisation

Professionals identified several factors influencing service organisation including the need for multidisciplinary/agency working, funding, local geography, waiting list management, service integration, and whether the service was needs-led or diagnosis-driven. At Integrated Site 1, the multiagency approach was facilitated by integrating various health and education services, for example, utilising educational psychologists to deliver an autism-specific diagnostic service, although the pathway was described as complex. They expanded,

. . . we have a complex pathway in that we have three different strands to the pathway, and that’s because we have two different CAMHS providers, which is a bit of a strange setting. And then we also have our pathway for the 0-5s separately which is not so strange I know, other areas have that. So that pathway is led by the paediatrician services in [XXX] Hospital Trust . . . . [Integrated 1. Project Manager]

Most teams had access to full-time MDT staff, but workforce recruitment and retention were often challenging. CAMHS Site 1 relied on part-time staff who joined the team intermittently creating barriers to effective team working. Commissioning arrangements were subject to frequent change, raising challenges around capacity, care pathways and funding. One-off time-limited funding (e.g., for short-term waiting list initiatives) was considered problematic, not least by commissioners, as it shifted challenges to other parts of the pathway. There was recognition that long-term, recurrent funding was much more helpful, allowing permanent solutions and recruitment.

One of the integrated sites successfully operated a joint CAMHS and CDT NDP. However, there was evidence of barriers to integration of CDTs with CAMHS, such as ‘tribalism, maintaining boundaries and separate histories’ (CAMHS 2 Commissioner). Another complexity of interagency working was for education practitioners who, despite delivering daily special educational interventions, were reliant on healthcare professionals (depending on location) to issue diagnoses before they could access additional funding or support. Challenges were identified for those whose diagnostic journey crossed age cut-offs between services. One commissioner (CDT 2) expressed frustration that once a child reached 5 years old, they would move from CDT to CAMHS but would start waiting again on a new pathway, without taking account of time already spent waiting for assessment.

While NICE guidelines call for a needs-led approach to diagnosis, many services focussed purely on diagnosis. Parents and teachers believed support and interventions were more likely to come with diagnosis. However, many parents experienced feeling ‘left to drown’, when discharged with no support following diagnosis. While access to timely autism assessment remains important, many interviewees suggested needs-based support should be available throughout the diagnostic process and not be autism-diagnosis dependent. With long waiting lists, a named point of contact was also considered important for parents and facilitated support.

#### PT4. Professional skill mix in assessment teams

All sites acknowledged that having a skilled MDT was essential for holistic assessment of possible autism and delivering appropriate interventions and support (where this was offered). The involvement of certain disciplines, beyond the core team suggested by NICE, was seen as beneficial including Learning Disability Nurses, Educational Psychologists, Early Years Practitioners, Portage practitioners and specialist HVs. However, professionals from all sites cited national shortages of core disciplines, affecting the ability to provide a multidisciplinary assessment:

. . . there’s a high turnover of staff, which impacts on morale. . .a new private sector service have nabbed some of our staff. . . they offer better benefits, pay etc . . . [CAMHS 2 Specialist Nurse]

One solution to increase diagnostic capacity was to utilise a wider range of professionals from different disciplines, often from less expensive roles, for example, a nurse specialist taking a developmental history instead of a doctor. This required staff to perform assessments based on their competencies rather than roles constrained by organisations and U.K. NHS Agenda for Change Banding (a national pay structure based on skills and experience). Families reported they would accept any professional undertaking the assessment if they were competent, and they valued the multidisciplinary approach:

I guess it’s important seeing different people, to see their point of view . . . it helps in getting more than one person’s point of view. [CAMHS 1 CYP]

For professionals, feeling valued created an environment where staff wanted to continue working within the service. Professionals thought flexible roles are more likely to be acceptable when teams work in a non-hierarchical manner. However, some teams maintained a hierarchical environment, for example, doctors would acknowledge other’s assessments but still make the diagnosis independently of the rest of the team (CAMHS 1, speech therapist).

#### PT5. Diagnostic assessment

The approach taken to diagnostic assessment and the views of interviewees varied between sites and individuals. Experienced clinicians often used clinical judgement and adapted diagnostic tools, such as proformas for diagnostic history taking. Less experienced practitioners were more likely to use standardised tools. Some parents mistrusted the reliance on algorithms with these tools. The study was carried out during the Covid pandemic, which resulted in the uptake of digital approaches facilitating socially distanced assessment, such as video-observation and online history taking. There were mixed responses to these approaches among all groups. Some CYP questioned how they could be diagnosed based on a 10-min online meeting; however, some parents liked receiving the diagnosis in the safety of their home. Young people suggested that choice was important:

. . . it’s just up to the child or what they would want. I’d prefer to just go there and do the exam and then just go home or just go out [CAMHS 1 CYP]

Some teams adopted a more flexible approach to assessment, where the components of assessment and professionals involved were tailored to the diagnostic complexity of the child. In some cases, this was facilitated by information gathered ahead of referral, determining who should be involved in each assessment. At two sites this contributed to being able to consider an autism diagnosis within a neurodevelopmental or mental health framework. However, most sites operated autism-specific pathways, requiring the child to subsequently enter a separate pathway to consider any comorbidities such as ADHD.

While MDT assessment was the norm, some practitioners would reach diagnosis independently, although relying on reports and observations from other professionals to inform decision-making. This required trust that was not always present, and that other professionals provide sufficient detail in their reports. Where this was the case, professionals commented that for complex cases verbal discussion was better than written reports so follow-up questions could be asked.

All sites used information from parents as part of the assessment process, including completed questionnaires. However, some parents found the questionnaires difficult to complete, and this was exacerbated if English was not the language read/spoken at home. Many parents and CYP found the assessment process patronising or confusing. When asked what it was like waiting to see someone to find out if you have this thing called autism, one child said:

Nervous . . . because I didn’t know what was going on really. [Integrated 2 CYP]

#### PT6. Feedback to parents and CYP and clinical reports

Feedback needed to be framed around the parent and CYPs needs and adapted to be sensitive to parents’ expectations and readiness. In some sites, feedback was combined with completing the assessment itself, which some parents found overwhelming. While they wanted feedback to be timely, they also needed to be ready to receive it. In a skill-mixed team, feedback may have only been given by one professional, leading to a perception that only this professional had been involved in assessing the CYP. Some CYP wished they had received diagnostic feedback directly and been able to see the report. However, one child commented about the report:

It wasn’t useful, it said a couple of things, but that was stuff I already knew, and it’s like fairly basic stuff, and not all of it applied to me, it was very general, and not at all like how I imagined a report. [Integrated 1 CYP]

Many parents felt abandoned after the diagnostic feedback session with no further opportunity to discuss implications at a follow-up appointment. Some sites would arrange a follow-up session if parents were distressed. Parents reported that it would have been helpful for a named professional to go through the conclusions with them after the feedback appointment. There was little evidence of ongoing support for parents following feedback.

Sites varied in the time taken to send out the diagnostic report. In one site, it was achieved within 2 weeks, but others took longer, potentially delaying access to appropriate interventions, although one site utilised an outreach team to work with CYP as soon as a diagnosis was confirmed.

Many sites did not routinely send diagnostic reports directly to the CYP’s school, leaving parents to do this. This lack of communication was frustrating for parents and school.

#### PT7. Training, service development and evaluation (maintaining and growing the workforce)

Professionals working within child development and mental health services regularly encountered CYP with autism, as did frontline staff in community-based CYP services such as schools and nurseries. However, training for frontline staff was often ad hoc, and multiagency training was limited. Many professionals, including Special Educational Needs Coordinators (SENCos), directly responsible for the care of children with autism in educational settings, gained most knowledge through experience and had little access to formal training. While availability of training at the local, regional, and national levels remained limited (even for core MDT disciplines), some respondents highlighted that the challenge was not the lack of training opportunities but the time to practise and integrate new skills into everyday work. However, one site recognised the benefit of offering in-house training in growing and retaining the MDT:

One of my support workers is a mum of a young lady with autism. So, I think we had people with really good skills, and we put them forward for training . . . I think if you grow your own you keep your own . . . . (Integrated Site 2, Manager)

#### Phase 3

At the end of the interview phase, the research team summarised the seven PTs into 27 recommendations for improving timeliness and quality of childhood autism diagnostic service delivery. These recommendations were presented to stakeholder delegates at dissemination workshops. Delegates ranked the recommendations and identified the 12 most important for service change and improvement (Table 4). Dissemination event delegates included around 250 clinicians and managers involved in diagnostic assessment (e.g., community paediatricians), parents/carers, commissioners, and academics. This led to final conclusions about the recommendations for those involved in commissioning, managing or participating in diagnostic services (Supplementary OS3 Figures 2 to 3).

**Table 4.** Recommendations for how to improve timeliness and quality of childhood autism diagnostic service delivery.

*1: Improve Recognition of Children Needing Referral*	**1. Develop system-wide awareness and increased professional recognition of ASD.** **2. Training on ASD recognition and meeting needs should be widely available and targeted at referrers.** 3. Designated NHS link roles to schools should be developed to liaise on interventions and referrals.
*2: Improve Quality of Referrals*	**1. Services need to gather information to inform decision-making over what assessment the child needs** 2. Needs to be a process in place to ensure that information at triage is up to date. 3. Need better tools for information gathering so professionals/parents can describe the child effectively.
*3: Service Organisation*	**1. When CYPs transfer from one service to another, the start date needs to be carried over to ensure that the CYP does not start at the bottom of the new waiting list.** **2. Co-existing conditions should be diagnosed alongside ASD under a neurodevelopmental umbrella.** **3. Need careful explanation of the service organisation, process and waiting list for parents, so they are fully prepared and understand what to expect.** 4. A (named) health professional should be a key person of contact, providing the family with continuity.
4: *Skill Mix and Effective Team Working*	**1. Staffing should be based on competencies rather than roles constrained by organisational banding. A skills-based team approach should be underpinned by intra-team trust and enhanced by staff retention, core team co-location, and non-hierarchical ways of working.** 2. Health professionals undertaking aspects of assessment should be part of the diagnosis decision meeting.
*5: Approaches to Improve Assessment*	1. Teams need flexibility to tailor the assessment according to diagnostic complexity, e.g., to undertake abbreviated (MDT) assessments where clinically indicated. This should include full assessment of strengths and needs; identify neurodevelopmental, mental health and co-existing conditions; and provide recommendations for ongoing support. **2. Choice must be given to parents and CYP about the use of digital. Face-to-face contact between CYP and a professional needs to be retained as an integral part of the assessment.** **3. Every effort should be made to adapt the assessment to the CYP’s age and developmental ability.** 4. If CYP are of sufficient intellectual capacity and want to be involved in the process, this should be facilitated. **5. If support is in parallel from the start of the assessment process, then the experience of parents and CYP feeling abandoned after diagnosis should be negated, and needs should be met.** 6. Where parents find completion of questionnaires challenging, then their named professional must talk through the questionnaire with them, and if needed, translation should be provided.
*6: Diagnostic Feedback and Reporting*	1. There should always be a follow-up session, preferably by someone known to family, maximising opportunities for parents to discuss what is important to them. 2. Where desired by CYP, feedback should be provided directly to them in an accessible way. 3. The report should be shared in a timely manner. Summary report may speed this process up. Adequate admin support is required, and tools such as dictation software may help. 4. With parental consent, reports should be shared with schools, so recommendations can be enacted. 5. Reports should be individualised, written in style appropriate for parents and CYP and translated if needed.
*7: Training, service development & evaluation (Maintaining and Growing the Workforce)*	**1. There needs to be access to training at local, regional and national levels, both generic for those who work with CYP who might have autism (e.g., Time for Autism, Oliver McGowan training) and specialist training for those developing autism diagnostic skills.** 2. Given an ageing workforce, succession planning needs to be prioritised. Need to enable senior staff to pass on their skills to less experienced members of the MDT. **3. Need to increase the numbers of staff trained in the core disciplines working in the MDT to increase the pool of people who could train as ASD diagnostic practitioners.**

Recommendations in bold were considered priorities at consultation workshops.

ASD: autism spectrum disorder; NHS: National Health Service; CYP: children and young people; MDT: multidisciplinary team.

## Discussion

Seven PTs were developed spanning the whole diagnostic pathway from identification, referral, assessment and feedback to report writing. Opportunities to improve services were identified within each PT, and recommendations for change developed during stakeholder consultation – some being implementable/low-cost and others needing additional resources (Table 3 and Supplementary OS5). In accordance with previous research (Morris, 2024; Russell et al., 2022), clinicians described rapidly increasing demand, and regardless of service model, the challenges of this with no additional funding for resources or time-limited waiting list initiative funding offering only temporary solutions. Despite guidance recommendations (NICE, 2011, 2014) and findings that MDT skill mix can improve assessment timeliness (Abrahamson et al., 2021; Parr et al., 2024), a lack of appropriately skilled MDT workforce and difficulties in recruitment and retention were evident.

Parents talked about ‘jumping through hoops’ to obtain a referral for diagnostic assessment and aligned with previous research being discharged with little support soon after diagnosis (Crane et al., 2016; Lappé et al., 2018; Rivard et al., 2023). For those parents where the referral process had taken years, their perspective of the diagnostic pathway aligned with previous research, being negative even where they acknowledged the diagnostic team positively (Makino et al., 2021; Rivard et al., 2021). All teams had implemented or planned adaptations to improve efficiency. Examples included effective information gathering, previously identified as helpful (Gordon-Lipkin et al., 2016) enabling triage; or being non-hierarchical, with team members’ skills and opinions given equal weight. Others introduced new disciplines within diagnostic teams including nurse specialists, or early years practitioners, focusing on competency over banding/professional background.

There was broad consensus among delegates consulted on the recommendations on challenges faced by services including imbalanced capacity and demand, one delegate describing recent increases in referrals as exponential. Consistent with previous research, there was recognition of the need for solutions and rethinking service delivery (Zwaigenbaum et al., 2021). Many delegates supported moving from condition-specific pathways to a neurodevelopmental approach (considering autism alongside co-occurring conditions, e.g., ADHD), and providing support for families throughout their diagnostic journey, not just at point of diagnosis. However, there were concerns about whether services could manage additional workload without additional resourcing. Teams interviewed shared experiences of attempting to move to a neurodevelopmental model – one describing success when given additional funding, while another without additional funding said it did not work. However, the findings highlighted some low-cost solutions, for example, improving the quality of referral information or diagnostic reporting.

A limitation of realist approaches is the potentially provisional nature of interpretations, given the theories are developed in the context of dynamic (rather than static) healthcare settings and organisations which are influenced by external factors, for example, political change (Pawson & Tilley, 2004). A limitation is the study being located in the U.K. NHS, with services funded through national taxation and free at the point of delivery which will impact the generalisability of the findings to other healthcare settings and contexts. However, many challenges and solutions identified are relevant to international innovation, for example, early support for families in India (Bhavnani et al., 2022; Divan et al., 2021) and single-practitioner assessment for children with very evident autism in Canada (Penner et al., 2023). Study strengths included considering how to improve autism service delivery through a realist lens, triangulating the views of service users, providers and those with lived experience to inform recommendations. Further strengths included identifying issues that were generally consistent across sites and representing a range of communities; differences between sites were not service model specific, for example, one CAMHS team ran autism-specific diagnostic pathways, while the other CAMHS team assessed autism within a neurodevelopmental and mental health context. A limitation is that where families had waited years to be referred, negative experiences may reflect older service delivery models.

Two sites utilised transdiagnostic NDPs; this is in accordance with developments in Scotland (Rutherford et al., 2021) and Wales (NHSW) and aligned with guidance that assessment systematically identifies neurodevelopmental, mental health conditions and other differentials (NHS Wales, n.d.; NICE, 2011). The findings highlight that focussing on CYP and families’ needs early in the assessment process should lead to better support from the start, rather than solely focussing on diagnosis. This should reduce referrals bouncing between services, and children completing multiple overlapping assessments for one condition, say autism, before the next, for example, ADHD, is considered. Benefits of delivering NDPs include integrating competencies of CAMHS and CDT staff (Parr et al., 2024) and cost and staff time efficiency (Male et al., 2020), although ensuring teams have the resources to deliver new models is required. The findings suggest integration beyond health, for example, with education can also help widen workforce skill mixes.

In accordance with previous research, the findings suggest education of potential referrers early in training, for example, the Time for Autism Program (Dhuga et al., 2022), coupled with enhanced information gathering (Gordon-Lipkin et al., 2016), could enable sufficiently detailed referrals to inform flexible assessments tailored to diagnostic complexity (Whitehouse et al., 2018; Zwaigenbaum et al., 2021). The findings suggest training programmes and supervision of emerging skill-mix clinicians are required to expand the workforce and facilitate early recognition and competencies-based non-hierarchical team approaches.

## Conclusion

Key areas for improvement were identified and summarised into recommendations that can be used to inform service development to improve timeliness, flexibility, efficiency, and quality of autism assessment services for CYP and families internationally. A key finding is that while making diagnoses remains important, support should not be diagnosis-dependent; rather, addressing the holistic needs of CYP and families from the start is paramount.

## Supplemental Material

sj-docx-1-aut-10.1177_13623613261430914 – Supplemental material for How can we improve the timeliness and quality of diagnostic assessment for children with possible autism? Qualitative findings and recommendations from a Realist Evaluation of Autism Service delivery in the United Kingdom Supplemental material, sj-docx-1-aut-10.1177_13623613261430914 for How can we improve the timeliness and quality of diagnostic assessment for children with possible autism? Qualitative findings and recommendations from a Realist Evaluation of Autism Service delivery in the United Kingdom by Ian Male, William Farr, Sophie McGrevey, Vanessa Abrahamson, Sarah Wigham, Venkat Reddy, Amanda Allard, Victoria Grahame, Jessica Maxwell, Grainne Saunders, Anna Walker, Nic King, Seema Islam, Zamir Akhtar, Jeremy Parr and Patricia Wilson in Autism
