# Supplementary material for: How can we improve the timeliness and quality of diagnostic assessment for children with possible autism? Qualitative findings and recommendations from a Realist Evaluation of Autism Service delivery in the United Kingdom
Source: Autism. 2026 May 10;30(6):1562–75. doi: 10.1177/13623613261430914 (PMC13187225; doi:10.1177/13623613261430914)
Supplement: sj-docx-1-aut-10.1177_13623613261430914 – Supplemental material for How can we improve the timeliness and quality of diagnostic assessment for children with possible autism? Qualitative findings and recommendations from a Realist Evaluation of Autism Service delivery in the United Kingdom [file sj-docx-1-aut-10.1177_13623613261430914.docx]

Online Supplementary Materials (OS) 1 A-D (for PTs see Table OS2)

| **OS1A Young People’s Interview questions** |  |  |
| --- | --- | --- |
| **Questions based on Programme Theories** | **PTs** | **Potential Follow on Questions to Dig Deeper** |
| **tell me a bit about…. [family/hobbies/school or whatever…]**  **Let’s start by thinking back to before you were seen by anyone about having a diagnosis like autism.**  Can you remember what (or who) started the process? Were you worried about something, or having any problems at home or school? | PT1  5-10mins | *Interviewer will have re-capped on purpose of interview before this first question*. |
| **What was it like waiting to see someone to find out if you had autism?** | PT2  As above mins | How did it make you feel? |
| **When you had the assessment(s), can you remember who you saw and what sort of things you did or talked about?**  **I’m going to ask you about some new ideas that are being tried out to see what you think of them** (ONLY IF *already discussed*):   1. Some autism teams are interested in using computer or ipad games to help test children who might have autism. What do you think about this? 2. Sometimes the team might talk to your parents so that you don’t have to carry out some of the tests. 3. What about using zoom or something similar, so that the autism team/doctor can watch you do some games or tests at home with your parents? 4. Some services mix and match who you see, like a doctor and speech therapist, and how many professionals you see at a time. What do you think about this? 5. Would you be happy with fewer visits to find out if you have autism? But we would not get to know you so well? | PT3  5mins | Don’t worry if you’re not sure about anything, just tell me what you can remember.   - Did you have to attend got to different places? - Did you understand why? - *Probe for* ***skill mix***: e.g. Did you know who was who, and what they do? E.g. speech therapist - *Probe for* ***model*:** see if can elicit who did what & mix of interviews, observations (e.g. in school, remote) and standardised assessments - *Probe for* ***digital innovations*** e.g was anything done remotely during covid lockdown? |
| **How did you find out the outcome of your assessment?**  **Can you think of any ways to improve how you were told?**  *If negative*: What would have been a better way of telling you?  **Did you feel that [the person who told them] explained the outcome?**  **Did you see anything written down, like a report?** Was if helpful?  **What did the assessment (and report) say that was important?** | P4  5mins | - Who told you about your diagnosis? i.e. 1 prof or MDT? - Straight away or later? (i.e. long wait?) - What was it like being told this? - Was there anything you didn’t understand (about the assessment itself, or the outcome)? - Could it have been made more understandable and useful? - Probe if looked at strengths as well as weaknesses |
| **Did you feel that people, including the doctor/nurse/therapist and your mum and or dad, listened to you at the appointments, and wanted to know how you felt?** Did you feel included in making decisions?  **While you were waiting to be assessed did you get any extra help, for example at school?** Did you have someone to talk to about the assessment?  **Did mum and or dad get any help? Did this change anything at home?**  **What happened after you had the diagnosis?**  Did anything change, for example, getting extra help at school?  What difference did this/these changes make? | PT5  5mins | - Probe who they felt listened and why. What was it about the clinician’s communication style that worked well? - Probe whether it was profs, parents or even CYP that made decisions. - Probe for any support pre, during or post-assessment. - Probe what help and whether directed at child or parents - Probe for follow up |
| **Did different people talk to each other about how to help you?** For example, did the autism team talk to your school?  **Some people think it would be a good idea to get parents and children to work with the autism teams to help improve services**. What do you think about that? How might that actually happen? | PT6  5mins | Probe if they have ideas on how they could be involved in service development and whether (for older children) they would want to be ‘separate’ to parental involvement. |
| **Once you knew you had a diagnosis, or not, did that help how you felt about yourself?**  **Some people say it may help to meet other children or young people with autism? Have you met any other children with autism? Was this helpful?**  **If one of your friends had to go through the same process what would you tell them about it? What was good about it? What didn’t you like?**  **Would you like to add any other ideas before we finish?** | Overall  5mins | - Has knowing you are/ not, autistic changed anything? - Would you have preferred to receive help without going through the assessment process? |

| **OS1B Parent Focus Group Topic Schedule** |  |  |
| --- | --- | --- |
| **Starter Questions based on Programme Theories** | **Which PTs** | **Potential Follow on Questions to Dig Deeper** |
| **Could you start by telling us what you hoped would be the benefits for your child of being assessed for possible autism?**  **What were your expectations? How have they changed?** Why did you feel that seeking a diagnosis was important? | Overarching  10min | Treat this an **introductory question** and keep a note of anything to come back to further down.  Probe if they had *specific expectations* (e.g. treatment, services) as that could have influenced how they engaged in the process.  Probe for how views have changed with *hindsight* e.g. I should have realised that…. (i.e. linking with follow up) |
| **What were your experiences when discussing your concerns with whoever you first approached**? What were your expectations?  **It has been suggested that when parents feel their concerns are taken seriously, for example by their GP, or any professional, this leads to timely referral to an appropriate service. Any thoughts about this?**  **How did this lead to referral?**  Do you know about local service thresholds and gateways to support?  What about your referral process worked well?  Were there any challenges?  Have you been offered any non-statutory services? e.g. voluntary led groups. Has this been helpful?  **What happened next?** (*this* *should cover length of wait, communication, being passed between different service (does it always get shared? How do you know?), being referred onto tertiary services, general dissatisfaction*) | PT1/2  10min | - Who did they discuss with and how often, over how long? E.g. school  - Did they feel their concerns listened to & acknowledged? *If not*, expand  - Did whoever they talked to appear to know how autism can manifest?  - Did they do their own research to persuade GP/other to refer?  *- If parent didn’t initiate the process:* ask them who raised concerns, how they felt about this & how it led to referral  - Who suggested referring? Did you have to ask more than once?   - How long did it take to get a referral? - *If didn’t initiate or know about autism*: did the [GP/referrer] explain who your child was being referred to and why? - Did anyone explain what would happen next? - How long did it take to hear from the service? - Did anyone keep you informed? Did you know who to contact? - Were you, or anyone else like your GP, asked to provide information? *Probe what information and how* e.g. online, paper form |
| **What were your experiences of going through the diagnostic process? What clinics or services did you go to?**  **How do you think your child found the process? And the family as a whole?**  **Who was involved in the assessment process?**  **Can you tell me about the format?**  *If not an integrated ND pathway ask*: **In some area services have an Integrated CAMHS/Child Development Neurodevelopmental Pathway, allowing a child to go through a single pathway looking at all their needs e.g. a child may have autism and ADHD. What do you think about this?**  *If no one mentions, ask:* **In some areas, teams have joined together to deliver autism diagnosis in a single centre. However, sometimes there is a trade-off between travelling a long way to a specialist centre and being seen quicker, or waiting to be seen locally. What do you think?** | PT3  35mins | Probe what type of service (model/assets based approach?), who they saw (skill mix), delays (single point of access?), onwards referrals (tertiary?) e.g. we’re interested here in pathway model, skill mix, points of access, clinical judgement, digital technology use   - Did you have to attend different local services to complete the assessment e.g. from CDC to CAMHS? - *If referred to specialist centre*: how hard or easy was this? Did you understand the reason for this? - *Probe for* ***skill mix***: e.g. Did you know who was who, and their roles? For example, was speech therapy involved? - *Probe for* ***model*:** see if can elicit who did what, how, where. Probe for interviews, observations (e.g. in school, remote) and standardised assessments. What was the balance between obs and other aspects e.g. clinical judgement standardised Ax. (note: parents may not be able to fully differentiate areas so could produce an overall perspective of the process) - *Probe for* ***digital innovations*** e.g was anything done remotely? (e.g. remote observation during covid) |
| **How were you told the outcomes of the assessment?**  Do you have any suggestions to improve this?  **How helpful was the final outcome?** e.g. diagnosis, no diagnosis?.  Did the diagnostic team consider other conditions or problems/needs? e.g. identifying other diagnoses beyond autism such as ADHD, anxiety  **Did you receive a written report?**  Could it have been provided in a more helpful format?  **Do you think the assessment and any reports you received understood the overall needs and strengths of your child, including any other conditions like ADHD?**  **Roughly how much did it cost to go through the process?** e.g. loss of earnings, access appropriate support, travel to clinic | PT4  15mins | - who gave feedback (individual or MDT?) - how e.g. in person or remote? with CYP? - when e.g. How long did you have to wait for it? - how they (and child) felt e.g. What was it like being told this?   *If no*: some area services have an Integrated CAMHS/Child Development Neurodevelopmental Pathway, allowing a child to go through a single pathway looking at all their needs e.g. a child may have autism and ADHD. What do you think about this? *If yes*: ask to elaborate   - What was helpful about it? Was it properly explained? Was there anything you didn’t understand or was a surprise? |
| **NHS England is concerned about the length of time it takes to complete a diagnostic assessment. Some services have introduced approaches to try and reduce waiting times. You may have experienced some of these approaches.** What do you think about the following ideas:   1. Assuming they are fully trained in autism assessment, would it matter to you what type of professional does the assessment? 2. Carrying out a shorter “Abbreviated assessment” with children who show a very clear autism picture? (*Was anyone aware of having an abbreviated assessment?)* 3. If the assessment or part of it was carried out by video observation and/or consultation? (*Did anyone experience this during lockdown?)* 4. Using digital technologies such as specially designed digital games or tools that can support diagnostic assessment? | PT3  15mins | *Aim to elicit views of parents who have actually experienced the innovation before gleaning general views*   1. For example, a speech therapist can do some parts of the assessment instead of a doctor. 2. This could free up time to see other children who need a more detailed assessment 3. During Covid lockdown, many teams have conducted clinics by video conferencing to work in safely & this could be used beyond Covid. 4. This could be an assessment of [e.g. emotion, facial recognition] on an i-pad or phone |
| **Can you tell us about any support you received, before, during or after the assessment?**  **What do you think “good” support should look like?**  **There is the idea that if parents understand the diagnostic process and feel supported this can improve their experience.** What’s your view on this?  **There is the idea that parents should be included in decision-making, as co-experts in the diagnostic process.**  What do you think about this?  Did you feel included in decision making?  **It has been suggested that parents and young people should work with professionals to influence service design. If you could influence the future design of autism diagnostic services what would you suggest?** | PT5  15min | - Were you referred to any support or training? - What was useful? - What did you expect to happen after completing the process? - What was helpful or unhelpful? [try to differentiate stage and exactly what e.g. what sort of info provided in what format] - Did you have a single point of contact/named key worker? - Did anyone explain the diagnostic pathway? - What would have helped you to feel supported? - What would have helped you feel included in making decisions? |
| **Did those involved in your child’s care appear to work together and talk to each other, as well as to you, while going through this process?** | PT6  10min | *They may refer back to PT2, being kept informed while awaiting assessment, but this is more extensive, for the whole process*.  Was your GP kept informed? Or the school? |
| **The last few questions are to help you (as a group) reflect on the process overall:**  **What worked well about your child’s journey through the assessment process?**  What do you think are the reasons for this?  What were the outcomes, in terms of, for example support?  Were there any negative or unexpected outcomes?  **What were the challenges you faced in going through the diagnostic process?**  **How would you describe the process to another family seeking a diagnosis for their child? What would you say to help them?**  Have you got any other comments or suggestions before we finish? | Overall  10min | - What was helpful about the process E.g. reassuring, affirmative ? - Were there beneficial outcomes, e.g. help with home life, schooling, accessing funding or further support? - E.g. length of process, number of appointments, travel, telling your story to different professionals, need for onward referral to specialist centre Cost in terms of loss of income isn't the only cost to be considered. There are additional costs such as emotional cost, cost to personal well-being which may also affect the young person, parent carers and wider family members. Have you anything to comment with regards this? |

| **Table OS1C. Clinician Interview Questions** |  |  |
| --- | --- | --- |
| **Starter Questions based on Programme Theories** | **Which PTs** | **Potential Follow on Questions to Dig Deeper** |
| What are the main features of your service? Or  How would you describe your service to other people?  What do you see as the purpose of your Autism/ Neurodevelopmental diagnostic assessment?  **Can you tell me about the size of your team and the skill mix, and any recent changes?**  **What is your individual role within the pathway?** | Intro  5min | *Probe to see if reason for changes e.g. to accommodate demand or recruitment/retention issues*  *If joint ND pathway, interviewee may refer to assessing co-occurring conditions which is under PT3* |
| **Who can refer into your autism/neurodevelopmental pathway?**  What are the criteria? (*research team may already know*)  **What referral information do you ask for?**  Do you provide guidelines or have a standardised referral form?  What is it about your referral process that works well?  What are the challenges? (*e.g. missing information*)  **Can you tell me about the triaging process please?** | PT2  10min | - Do you offer training to potential referrers about autism (or other) conditions? *If yes, probe for more information* - Have you been able to improve the quality of information available at the time of referral? Has this made triaging easier? Does it save time? Do fewer children go through the full process? - Do you seek additional information? e.g. questionnaires, screening tools like M-CHAT-R, or observations? *Probe who from, how & any problems* - Prior to the referral do you expect the child to have received support and/or assessment by community services-e.g. Early Help, behaviour programs such as Triple P, Solihull? - Can you expand? What is it about triaging that enables [whatever it is they just said]? - How have you addressed capacity issues? |
| **Can you tell me about your autism diagnostic assessment process and what you think works well?**  **How would you describe the approach?**  **Do you offer different approaches according to complexity of presentation?**  **What innovations did you introduce during covid-19 lockdown? Which of these do you intend to continue with?**  **What is your approach to assessing co-occurring conditions, for example, ADHD or anxiety? and underlying conditions e.g. genetic, Developmental Trauma**  **How is this funded or commissioned? Are there problems?**  ***Or:*** we’ve already mentioned that you can assess co-occurring conditions, can you tell me in more detail how this works?  **As I am sure you are aware, NHS England have concerns over the length of time it takes to complete a diagnostic assessment.**  **Has this been an issue? How has your service addressed it?**  **It has been suggested that service configuration, e.g. skill mix can impact upon effectiveness or capacity. Thinking about your service, can you comment on this?**  **There has been a lot of interest in developing integrated neurodevelopmental pathways working across CAMHS and Child Development Services, and potentially other agencies.**   - *For teams using an integrated approach*: **what has enabled this to work well? Have there been any barriers and how did you overcome them?** - *For those not yet integrated*: **what do you think about an integrated pathway? Can you see any barriers to establishing one? What might help?** | PT3  25mins | - Do you use **skill mix** approaches at all? How you do this? - Is skill mix affected by problems **recruiting and retaining** staff? - Probe for balance of **standardised** tools, **observation** (what/ when), **interview**s with parents, **clinical judgement**. - Do you use any **digital technologies** to assist in your assessment e.g. QB Test? Video observation? - *Looking for if they use an assets-based approach (try not to use this term however)* - What challenges have you encountered? - What have you had to do to make these approaches work? - e.g. “abbreviated” assessment for so called “barn door” cases - What worked well? Or not? *Ask to expand on reasons* - What is the process if you think a child needs assessment for co-occurring conditions? Is this within the team? - *If not*: who do you have access to, e.g. child psychiatry? - *If not*: what is the process for referring to other services? Are there any problems doing this, like funding?      - How are staff shortages managed? Some services have adjusted who does what, according to local availability e.g. speech therapists completing interview instead of doctor. - *Probably already covered, but might be more to probe* |
| **How do you feedback the results of the assessment?**  **What is the focus?** (*looking for assets based approach*)  **Can you tell me about the report, for example, the format?** | PT4  10mins | - Who gives feedback (individual or MDT?) - How long do family usually have to wait? - What is the purpose of this report? - In your feedback and report writing what if anything do you consider in addition to scope of the diagnosis of autism or not? - Is report format same for everyone? (*probe to see if they use assets-based approach*). Who is it sent to? |
| **What support do you offer to families going through assessment?**  **What support is available after diagnosis/beyond completion of the diagnostic pathway?**  **What do you think “good” support should look like?**  **There is the idea that if parents understand the diagnostic process and feel supported this can moderate their expectations.** What do you think about this idea?  **There is the idea that parents should be included in decision-making, as co-experts in the diagnostic process. What do you think about this?**  **There is the idea that parents find it helpful to have a single point of contact – does your service offer this?** | PT5  10mins | - What do you see as the value of offering support, during the diagnostic process? - Is this dependent on diagnostic outcome? What support do you offer to families where their child does not receive a diagnosis? - Do you offer or refer to any specific support or training packages? Or run any groups during time on pathway? - Do you offer parents a named contact, key worker or equivalent to support them through the diagnostic pathway? - At what level would this be suitable? Probe for admin/first point of contact role in pathway (however try not to suggest admin as key worker) |
| **What issues are there around inter-agency or inter-professional working?**  **What initiatives has your service undertaken to promote integrated working at a strategic level?** | PT6  5mins | - *Individual level refers to integrated working for specific children e.g. talking to their school* - *Strategic level refers to policy/other initiatives to promote integrated working between health, social care and education* |
| **The next questions relate to training, service evaluation & development.**  **How do you think your service has been affected by commissioning decisions, either recent or historical?**  **What training are you and your colleagues able to access to enhance your skills around autism and related disorders?**  **There is an idea that introducing skill mix approaches may help increase capacity for diagnostic services? (***May have already covered)*  **How have you, or would you, support and supervise the development of staff in such skill mix roles? Has anything helped or hindered this?**  **Might this help with recruiting and retaining staff?**  **Does your service provide any training to upskill staff who work with children in generic/community settings and might be able to identify those needing assessment, with suitable training? (***May have already covered under PT2)*  **It has been suggested that the use of audit / service evaluation / quality improvement approaches may help find solutions to pathway delivery. What has your experience been in your service?** | PT7  10mins | - Does your Trust support you to attend such training? - Have nurses/AHPs seeking to take on specialist roles been able to access relevant training e.g. Masters - Have you developed in house approaches that you would be happy to share? What does this look like? - Is there any training *across* services to improve inter-professional working? - *If not used, why is that?* |
| **Finally, if you had to summarise what it is about your autism diagnostic assessment process that works well, what would you say? What do you think are the key reasons for this?**  **If you had access to more resources (staff, funding etc) what would you change?**  **Is there anything else you would like to tell me about your service that we haven’t already covered?** | Overarching  5mins | - How, if you have done so, have you addressed any challenges? |

| **Table OS1D. Referrer Questions** |  |  |
| --- | --- | --- |
| **Starter Questions based on Programme Theories** | **Which PTs** | **Potential Follow on Questions to Dig Deeper** |
| **What is your individual role in supporting children and families?**  **Can you tell me a little about your experience, training or involvement with children who have, or may have, autism?** | Intro  5mins | - Or role within child’s care pathway |
| **How confident are you in recognising the signs & symptoms of autism?**  **Have you had any training (post-qualifying) on recognising the signs and symptoms of autism & related conditions?**  **What expectations do you have when referring a child for Autism / Neurodevelopmental diagnostic assessment?**  Prior to referral, do you have access to any support and/or assessment by any other service or organisation? | PT1  10mins | - *If interviewee mentions training, ask this. If not, ask under* ***PT7*** - What do you see as the potential benefits for the child/family? - e.g. Early Help, behaviour programs such as Triple P, Solihull? |
| **How confident are you in deciding whether a child needs referral for an autism assessment?**  What would prompt you to refer? Or to ‘wait and see’?  Who else might you discuss this with?  **Where would you refer if you suspect the child has other conditions as well as autism? Have you encountered any difficulties in doing so?**  **There’s the idea that providing clear guidelines for referrers on what information is needed & how to refer saves time, enabling better selection of which children access the diagnostic service. What are your experiences of this?**  **It has also been suggested that training potential referrers may improve the quality and appropriateness of referrals. Have you had any training or guidance in knowing where to refer, service criteria and what information to provide?**  **Many referrers and families complain that referrals bounce backwards and forwards between for example CAMHS and CDC. Have you experienced this?**  ***If have a single point of access or integrated service (which we should know in advance):* what is helpful about this?**  ***If not*: It has been suggested that a single point of access, or an integrated CAMHS/CDC neurodevelopmental service, makes the referral process more efficient. Do you have any thoughts about, or experience of this?**  **Is there anything specific about the referral process that gives you confidence in the service provider?** | PT2  10min | - If a parent asks you to refer their child, do you use any questions, screening tools or questionnaires to help you decide? Would you be happy to use a screening tool? - *Probe for cross-disciplinary/organisational working* - *Probe for rationale* - For example, *dyslexia, ADHD or anxiety disorder*   What would help?   - Are you aware of the triaging process? - Have you used standard referral forms – are these helpful? - What challenges have you encountered/do you foresee? - Do you seek additional information to support referral e.g. questionnaires, recognised screening tools like M-CHAT-R, or observations? Who do you ask for this information? - Depending on answer to what works well could follow up with deeper questioning, e.g.:  *So are you saying that access to screening tools is an important aspect of ensuring the right children are referred at the right time? OR ‘what is it about using screening that ensures….’* - Can you think what would help address this locally? - *If they mention single point of access, ask to expand* |
| **What is it about your local autism diagnostic assessment service that works well? What do you think are the key reasons?**  What feedback have you had from parents about the process?  **If you could, what would you like to change about the service?**  **What challenges have you faced in using the service?**  **NHS England have raised concerns over the length of time it takes to complete a diagnostic assessment. What has your experience been?**  **Are you aware of any approaches your local providers have introduced that have helped speed up or improve the process?**  *For education professionals*: **What roles do you think that teachers, EPs or similar could take in the diagnostic process?**  **There has been a lot of interest in developing integrated neurodevelopmental pathways working across CAMHS and Child development services, and potentially other agencies such as education. What do you think might be the benefits or disadvantages?** | PT3/  overarching | - How have you tried to address any challenges? - Have you been aware of changes in the experience for children you have referred e.g. change in waiting times or criteria? - e.g. remote assessments or alternative arrangements during the Covid-19 pandemic? - What would you think if the service offered different approaches according to complexity of presentation, e.g. “abbreviated” assessment for so called “barn door” cases - Are you aware if they use skill mix approaches? - Do they use any digital technologies to assist in the assessment e.g. QB Test? Video observation? - How easy would it be to use video observation in your school or pre-school setting? - **Have you had any experience of this (elsewhere)?** |
| **How do you get feedback about the assessment?**  **Can you tell me about the report, what’s helpful, what’s missing?**  **Do you discuss it with the family or anyone else involved in the child’s care?**  **It has been suggested one outcome from diagnostic assessment should be to create an accurate picture of a child’s strengths & needs to inform individualised packages of support & intervention. What’s your view, and experience, of this?** | PT4  PT4/6  PT3/4 | - *Prompt re how long they have to wait for report* - Is it understandable/useful? i.e. does it help you figure out what to do next? - What information/guidance would be more helpful? *Link back to what they said their expectations for assessment were*. - What difference would this make? |
| **What support (if any) do you offer to families going through assessment, and beyond completion of the diagnostic pathway?**  **Do you expect the diagnostic service to provide support while awaiting assessment, during or after the process?**  **What do you think is feasible, irrespective of getting a diagnosis?**  **There is the idea that parents, and schools should be included in decision-making, as co-experts in the diagnostic process. What do you think? What’s your experience been?** | PT5 | - Is this part of your role? - What support do you offer to families where their child does not receive a diagnosis? - *Ask what their experience is* - *We appreciate that some children clearly need assessment and funding would be an issue, but we are interested because parents may seek diagnosis because they view it as a gateway to services [might have come up under PT1-2]* - *Probe their interpretation of co-expert* |
| **What has your experience been of working with your local autism/ neurodevelopmental diagnostic services?**  **What would make it easier to work more closely across agencies and professional disciplines?**  *For school staff*: **Do you feel the views of school staff are considered in the diagnostic process/outcome?** | PT6 | - What works well? *Probe who/how* - What have been, or might be barriers to joint working? - How have/could you overcome these barriers? - What formal channels are there to promote integrated working at an individual or service level? - For example, some services observe the child in school and discuss with teaching staff. What has your experience of this been? How would you feel about this happening in your school? |
| **What training are you and your colleagues able to access to enhance your knowledge around autism and related disorders?**  **Does your organisation readily support you to attend such training?**  Is there any local inter-organisational or inter-disciplinary training?  Are there any current service developments related to the autism diagnostic pathway that you are aware of or involved in? | PT7  PT6/7 | - Have you developed in house approaches to training that you would be happy to share-what does this look like? - How easy would it be for you to attend such training if it were available? Or does your organisation support you to attend training? - How much of an issue is cost of training-e.g. will your organisation pay for you to attend or allow you the time? - Probe for service evaluation or service developments that might relate to diagnostic pathway |
| **Is there anything else you would like to discuss that we haven’t already covered?** |  |  |

| Table OS2. Initial Programme Theories (IPTs) guiding the interview questions  Overarching PT: If there is a MDT assessment by a team with competencies in child neurodevelopment & mental health (context), then autism will be recognised as a complex condition that relies on detailed history & observation across settings (mechanism) to diagnose it. This will lead to accurate diagnosis, recognition of associated co-occurring conditions such as ADHD & intellectual disability (outcome), & the ruling out of complex differential diagnoses. This will also create, whilst not an explicit part of this project, an accurate picture of a child’s strengths & needs to inform individualised packages of support & intervention through health, education & social care (outcome). | | |
| --- | --- | --- |
| PT 1: Recognition   1. Parents/carers concerns listened to & discussed 2. Frontline health & education professionals are cognisant of ASD & referral pathways | If frontline health & education professionals (e.g. GPs, teachers) are confident in recognising the signs & symptoms of autism, are cognisant of referral pathways & take parents/carers’ concerns seriously then CYP will be referred to the appropriate service, in a timely manner, reducing parental frustration. |  |
| PT2: Referral   1. Referral process 2. Triage | If Autism diagnostic services provide clear guidelines for referrers on what information is needed & how to refer, then time will be saved at the triaging stage & fewer CYP who do not have Autism will go through the full process. |  |
| PT3: Diagnostic model   1. Model & skills mix 2. Clinical judgement 3. Digital technology | There is wide variation in the model for Autism diagnostic services & national staff shortages but these can be addressed with a structured & consistent approach, making best use of available staff & clinical expertise. | ***asset-based approaches*** *to diagnosis & support in autism; the use of* ***digital technology*** *in diagnostic assessment.* |
| PT4: Diagnosis & support   1. Diagnostic feedback to parents & CYP 2. Report format | If parents understand the diagnostic process & feel supported this can moderate parental expectations. Feedback should take an assets-based approach & the management plan should be individualised, taking account of co-existing conditions. Reports should be timely & in a format that everyone finds helpful. | *barriers & facilitators to comprehensive* ***needs-led*** *diagnostic assessment.* |
| PT5: Working in partnership with families   1. Parent/carer as co-experts in the diagnostic process 2. Supporting parents/carers | Parents find the diagnostic pathway stressful so find it helpful to have a single point of contact; to be provided with explanations about the process; & to be included in decision-making. |  |
| PT6: Inter-agency working   1. Macro-Meso level 2. Micro level | If “experts” including people with Autism, carers, professionals & specialist organisations work in partnership & the knowledge generated is effectively embedded into local services, this will build capacity, improve parent/CYP satisfaction & support planning of services both locally & nationally. | *approaches to* ***integrating services*** *dealing with autism.* |
| PT7: Training, service evaluation & development   1. Training for professionals working with CYP in community settings 2. Training for health professionals working in Autism services 3. Service development & evaluation | Based on their needs, skills & knowledge for Autism diagnostic assessments & working with families, health & community professionals should have access to tailored training, service development & service evaluation. | *training & support materials available for* ***non-specialist*** *staff &* ***parents & CYP*** *accessing the service.*  *training packages for* ***those working in autism services*** *to upskill, & evaluation of the impact on* ***workforce shortages****.* |

|  |
| --- |
| **OS3. Figure 1. Key Questions presented at national consultation meetings** |

**OS3 Figure 2. Key conclusions presented at national consultation meetings**

|  |
| --- |
| OS3 Figure 3. Key conclusions presented at national consultation meetings |

| **Online Supplementary Materials 4 (OS4):** Programme Theories (PT) | |
| --- | --- |
| PT1. Recognition | If potential referrers such as GPs, are trained to recognise symptoms suggesting possible autism, and listen to parents’ concerns, enabling them to refer in a timely manner, then this can speed up the diagnostic journey, and improve the diagnostic experience for the child and family. |
| PT2. Referral | If detailed information describing reasons for concern and the child’s strengths and needs, is sought at the time of referral, then this facilitates decision making as to whether the child needs to be seen, and what assessment is needed, for example enabling early referral to alternative support or diagnostic pathways. |
| PT3. Service Organisation | If services including CAMHS and CDT work together offering access to multidisciplinary competencies each service brings, then it is possible to deliver a diagnostic assessment that considers the child’s strengths and needs, and a neurodevelopmental/mental health diagnostic formulation, which avoids referrals bouncing between services. Focus on recognising needs within the assessment, alongside diagnosis, can lead to early and sustained support for child and family. |
| PT4. Skill Mix | If there is a non-hierarchical, multidisciplinary team that recognises and utilises the competencies of its staff, regardless of grade and job title, then it is possible to deliver a more flexible approach to assessment, with a wider diagnostic and support workforce, increasing the capacity of the service to meet the demand for diagnostic assessment. |
| PT5. Assessment | If there is a flexible approach to assessment that is responsive to the complexity of the individual child’s presentation, and open to new approaches to assessment, e.g. digital solutions, then it may be possible to increase service capacity, and focus on early support for the child and family. |
| PT6. Feedback and Reporting | If reports, including a description of the child’s strengths and needs alongside a neurodevelopmental diagnostic formulation, are written in a format accessible to parent, and CYP, and they have the opportunity to discuss this with a member of the diagnostic service, and if, given parental consent, this is made available to the child’s educational setting, then this can inform those delivering care to the child to better understand how to meet the child’s needs. |
| PT7. Training | If training is provided for potential referrers to be able to recognise early warning signs of autism, e.g., teacher training, medical students, and for those working in, or who could become part of diagnostic services, then this could increase diagnostic service capacity and timely access to diagnostic assessment. |

| OS5. Quotations Related to Programme Theories | |
| --- | --- |
| PT1: Recognition | *'It went undetected, so I always thought the problem was my attitude, or something. I just think that I always felt like I was the problem…*' [Integrated Site 1, CYP]  *'It was Year 5 he had an amazing teacher that had him for one term and she went to me, ”I don’t mean to be rude but have you ever thought that he’s on the spectrum”? And I’m like thank you, at last….'* [Integrated Site 2, Parent]  *'...I think the nursery listened to our concerns. I’d spoken to the health visitor about the fact that he wasn’t talking and his two-year check as well, I kind of relayed my concerns to them and they listened...'* [CDC Site 2, Parent] |
| PT2: Referral | *'I got a letter back saying that they couldn’t accept the referral… I’ve since found out that the doctor’s report wasn’t detailed enough, which made me quite angry*…' [CAMHS Site 1, Parent]  '…*he used the old primary school report... from before he started taking ADHD medication when he was a completely different child…*' [CAMHS Site 1, Parent]  *'Parents, said, “You’re trying to make us jump through hoops so we can’t access any assessment”… Afterwards “parents said “Actually, it was really good and, I got something out of it”… A year later - 30% didn’t want an assessment after they’d done the parenting support [from the Early Help Service]*.' [Integrated Site 2, Team Manager]  *'To avoid referrals “bouncing” between services: …If a colleague is very experienced in autism, there’s a… (appointment) where somebody will join a core autism team member and do a neuro-developmental-focussed assessment.*' [Integrated Site 2, Consultant Psychiatrist]  *'We gave score for complexity, to decide skill mix and assessment appropriate to needs of child…so children who are very obvious may not need same level of assessment'* [CDC Site 1, Paediatrician] |
| PT3: Service Organisation | *'We have a little bit of a complex pathway with two different CAMHS providers, and our pathway for 0 to 5s which is led by the paediatric services'* [Integrated Site 1, Project Manager]  *'There are lots of operational issues that the team is really virtual. I don’t think it works well…there’s so little time we can’t really gel as a team'* [CAMHS Site 1, Child Psychiatrist]  '…*we have non-recurrent monies, it often needs to be to be spent by March, but to recruit staff, it takes three to four months …it’s extremely difficult to do any long-term planning*.' [CAMHS Site 2, Commissioner] |
| PT4: Skill Mix | *'It’s not easy to recruit… and people go on maternity leave, there’s a continuous turnover of staff…it’s a challenge*…' [CDC Site 1, Paediatrician]  '…*speech and language therapists (SALT) see children week in week out, but their reports don’t hold as much weight as Educational psychologists who … only do initial consultations with parents and school…'* [Integrated Site 1, SENCo]  *'We look at who could do ADOSs or … ADIs…. That means the clinic can run regularly, and people can be slotted into those clinics as they come up'* [Integrated Site 2, Manager]  *'Our support workers do a lot of the behavioural assessments… there’s a general ethos of people getting extra skills and being trained up… The vast majority of the BOSAs are done by two support workers*.' [Integrated Site 2, Psychiatrist] |
| PT5: Assessment | '…*if at triage we already have a clear-cut parental history and there’s a play-based assessment, we’ll streamline the process for one final appointment where we pull it together and give a diagnosis*…' [CAMHS Site 1, SALT]  '… *I didn’t have chance or capacity to question the algorithm if you know what I mean?'* [Integrated Site 1, Parent]  '…*More than one parent said "I’m so glad this is on video" or "I don’t have to walk through the waiting room in tears now.”*' [CAMHS Site 1, SALT]  '…*we had to do a video appointment so they couldn’t see all the shaking and the fidgeting*…” [Integrated Site 1, Parent]  '…*he was talking to me a bit childishly… I thought that he was talking to me as if I was younger*…' [CAMHS Site 1, CYP] |
| PT6: Feedback and Reporting | '…*a formal letter [should be] sent, this is what they’ve been diagnosed with, the behaviours, what they need and the school need to provide for this child to succeed* …' [Integrated Site 1, Parent]  *'I wish I’d seen the report, but… I don’t really wish anything had changed about my mum telling me...*' [Integrated Site 1, CYP] |
| PT7: Training | '…*I have done a little training when it came to the SENCo qualification, but it was quite limited, and it was more about what our role is. I think there is a lack of understanding of what autism might look like'* [Integrated Site 1, SENCo] |
